# Genome Sequence of the Asiatic Species *Borrelia persica*

**DOI:** 10.1128/genomeA.01127-13

**Published:** 2014-01-09

**Authors:** Haitham Elbir, Pär Larsson, Johan Normark, Mukunda Upreti, Edward Korenberg, Christer Larsson, Sven Bergström

**Affiliations:** Department of Molecular Biology, Laboratory for Molecular Infection Medicine Sweden (MIMS), Umeå Centre for Microbial Research (UCMR), Umeå University, Umeå, Swedena; Division of CBRN Security and Defence, FOI Swedish Defence Research Agency, Umeå, Swedenb; Gamaleya Research Institute for Epidemiology and Microbiology, Russian Academy of Medical Sciences, Moscow, Russian Federationc

## Abstract

We report the complete genome sequence of *Borrelia persica*, the causative agent of tick-borne relapsing fever borreliosis on the Asian continent. Its genome of 1,784,979 bp contains 1,850 open reading frames, three ribosomal RNAs, and 32 tRNAs. One clustered regularly interspaced short palindromic repeat (CRISPR) was detected.

## GENOME ANNOUNCEMENT

*Borrelia persica* is the agent of relapsing fever (RF) in central Asia and Middle Eastern countries, but lack of awareness about the disease probably has led to an underestimation of its prevalence (1). Until now, *B. persica* has eluded *in vitro* cultivation, impeding its full characterization and *in vitro* experiments.

The *B. persica* strain No14 was isolated from *Ornithodoros papillipes* collected near the city of Khiva, Uzbekistan. The strain was isolated and propagated at the Laboratory of Vectors of Infections, Gamaleya Research Institute, Moscow, Russia, by passaging in guinea pigs (2). It was initially kept alive in a tick-guinea pig cycle and in 1988 it was stored at –70°C. After thawing in 2008, the strain was refractory to growth *in vitro* and was therefore propagated in CB17/Icr-Prkdc^scid^/IcrIcoCrl mice (Taconic, Denmark) and subsequently successfully grown at 37°C in freshly made Barbour-Stoenner-Kelly II (BSK-II) medium with 1.4% (wt/vol) gelatin and 10% (vol/vol) rabbit serum (3). DNA was extracted using the Wizard genomic DNA purification kit (Promega Biotech AB, Sweden) and sequenced using an Illumina HiSeq 2000 sequencer. *De novo* assembly of reads was performed with the ABySS 1.3.4 assembler, and open reading frames (ORFs) were identified with Prodigal (4). tRNAs were predicted with the Aragorn software (5), and ribosomal RNAs were predicted with RNAmmer. The genes were further annotated using BLAST against the NCBI nonredundant database. The functional categorization of ORFs was performed using online RPS-BLAST against the cluster of orthologous groups (COG) (6) and Pfam databases (7). PHAST (8) and the Prophage Finder software were used for bacteriophage detection (9). Clustered regularly interspaced short palindromic repeats (CRISPRs) were predicted using the CRISPRfinder server (http://crispr.u-psud.fr/Server/). To estimate the similarity with published RF *Borrelia* genomes (10, 11), the average nucleotide identity (ANI) was calculated as described in Konstantinidis et al. (12).

A total of 1,795,421 bp of sequence was generated from 15,331,580 reads with 826-fold coverage and a G+C content of 28.7%. The scaffolds were ordered, resulting in a linear chromosome with a size of 923,419 bp and unclosed plasmids with a size of 872,002 bp. Gene finding resulted in 1,850 ORFs, three ribosomal RNAs, and 32 transfer RNAs, which is similar to the genomes of other RF species (10, 11). The protein-coding regions represent 81% of the *B. persica* genome. Among the predicted genes, 43.4% were assigned a COG function. One CRISPR with three spacers was found. Comparing the chromosome of *B. persica* to the previously published genomes of *Borrelia duttonii*, *Borrelia recurrentis*, and *Borrelia crocidurae* revealed a high colinearity, with an average nucleotide identity of 89%.

Several genome sequences of African RF *Borrelia* spp. have been published (10, 11). The added *B. persica* genome sequence is of great importance as the first representative of Asian tick-borne RF borreliosis. An analysis of its genome sequence will provide further insights into its functional genomics and host adaptation. It will also facilitate future molecular and medical research on *B. persica*, a bacterium causing human disease, about which very little is known.

### Nucleotide sequence accession numbers.

This whole-genome shotgun project has been deposited at DDBJ/EMBL/GenBank under the accession no. AYOT00000000. The version described in this paper is version AYOT01000000.
